# The Availability of Slow and Fast Calories in the Dutch Diet: The Current Situation and Opportunities for Interventions

**DOI:** 10.3390/foods6100087

**Published:** 2017-10-02

**Authors:** Janet van den Boer, Melanie Werts, Els Siebelink, Cees de Graaf, Monica Mars

**Affiliations:** Division of Human Nutrition, Wageningen University, 6700AA Wageningen, The Netherlands; janet.vandenboer@wur.nl (J.v.d.B.); melanie.werts@wur.nl (M.W.); els.siebelink@wur.nl (E.S.); kees.degraaf@wur.nl (C.d.G.)

**Keywords:** oral processing, ingestion behavior, microstructure of eating, speed, energy density, food form, satiety, weight management

## Abstract

Choosing foods that require more time to consume and have a low energy density might constitute an effective strategy to control energy intake, because of their satiating capacity. The current study assessed the eating rate of Dutch food, and investigated the associations between eating rate and other food properties. We also explored the opportunities for a diet with a low energy intake rate (kJ/min). Laboratory data on the eating rate of 240 foods—representing the whole Dutch diet—was obtained. The results show a wide variation in both eating rate (from 2 g/min for rice waffle to 641 g/min for apple juice) and energy intake rate (from 0 kJ/min (0 kcal/min) for water to 1766 kJ/min (422 kcal/min) for chocolate milk). Eating rate was lower when foods were more solid. Moreover, eating rate was positively associated with water content and inversely with energy density. Energy intake rate differed substantially between and within food groups, demonstrating that the available foods provide opportunities for selecting alternatives with a lower energy intake rate. These findings offer guidance when selecting foods to reduce energy intake.

## 1. Introduction

Choosing foods that require more time to consume (i.e., foods with a low eating rate) might constitute an effective strategy to control energy intake [1,2,3]. Experimental studies have consistently shown that food (g) and energy (kJ) intake can be altered by manipulating eating rate (e.g., [3,4]). Moreover, literature indicates that a high eating rate is associated with a higher body mass index (BMI) [5,6]. It is hypothesized that when calories pass quickly through the oral cavity they do not bring about an adequate satiety response, resulting in an increased food and energy intake and eventually a higher BMI [7,8,9,10].

How quickly calories pass through the oral cavity also depends on the energy density of foods, which is another well-established predictor of energy intake [11,12]. Multiplying the eating rate with the energy density of foods is therefore expected to result in an even a stronger predictor of energy intake: “energy intake rate” (kJ/min). A recent experiment by McCrickerd, et al. [13] investigated the combined and separate effect of manipulating the eating rate and energy density of foods on energy intake using a 2 × 2 design. Their results show that the combined manipulation (i.e., rice porridge with a low eating rate and low energy density) is more effective at reducing energy intake than the individual manipulations alone (i.e., rice porridge with either a low eating rate, or a low energy density). These results demonstrate the added value of energy intake rate.

There is, however, limited information available on the eating rate—and therefore energy intake rate—of commonly consumed foods. Most studies that report the eating rate of foods involved manipulated or model foods (e.g., [14,15,16]). To our knowledge there are a few studies that have measured the eating rate of commonly consumed foods [2,3,17,18]. Viskaal-van Dongen et al. [3] measured the eating rate of 45 foods commonly consumed in the Netherlands. Forde et al. [17] measured the eating rate of 35 solid, savory meal components. Ferriday et al. [18] measured the eating rate of 20 different commercially available pre-packaged meals. Finally, Forde et al. [2] measured the food-specific eating rate of 47 commonly consumed Singaporean foods. These datasets, although substantial, do not represent a whole diet, nor did they consider the energy intake rate of the foods. Therefore the first aim of the current study was to assess the eating rate and energy intake rate of the foods commonly consumed in The Netherlands.

The second aim was to map the characteristics of slow and fast foods by investigating the associations between food-specific eating rate and other food properties (i.e., texture, food composition, and taste) in the obtained dataset. Moreover, this will provide more insight in the characteristics of slow and fast calories. Based on the literature solids were expected to have a lower eating rate when the texture is harder and drier, and liquids were expected to have a lower eating rate when viscosity is increased [15,16,19]. With regards to food composition, water content was expected to be positively associated with eating rate, while energy density and fiber content were expected to be negatively associated with eating rate [2,3]. Regarding taste eating, rate was expected to be inversely associated with taste intensity [2,20].

The third aim was to explore the opportunities for choosing foods with a low energy intake rate within the limits of the current Dutch diet. To this aim we tried to identify groups of food that differed in energy intake rate, within food groups, hereby investigating the possibility to vary in energy intake rate within the Dutch diet. Previous studies have indicated that the available foods will provide variation in both eating rate and energy density [1,2,3,18]. This suggests that it might be possible to design a diet low in energy intake rate given the available variation in energy intake rate.

Summarizing, the current paper will provide new insights regarding the eating rate and energy intake rate of commonly consumed foods. It will show (1) the variation in eating rate and energy intake rate across the Dutch diet, (2) the characteristics of slow and fast foods, and (3) the variation in energy intake rate present within food groups. This information could serve as a starting point when designing an intervention to reduce energy intake through the selection of foods with a low energy intake rate.

## 2. Materials and Methods

A database was built with the eating rate of foods representing the whole Dutch diet (Table S1). First laboratory measurements were performed to obtain the eating rate of 192 foods. This dataset was then expanded with existing data on the eating rate 48 foods. Finally, information on texture, food composition and taste were added.

### 2.1. Building the Eating Rate Database

#### 2.1.1. Laboratory Measurements

The eating rate of 192 foods was assessed in a laboratory setting. Every food was eaten by at least four participants while the time spent eating and amount eaten was recorded. Furthermore, three reference foods—eaten twice by all participants—were included to correct the eating rate data for the personal eating rate of the participants.

##### Foods

The foods were selected to reflect the Dutch diet with the help of a research dietician. To arrive at this selection the following aspects were considered: contribution to energy intake of Dutch population [21], representation of the different food groups, variation in texture, taste and macronutrient composition, and representation of different eating occasions (e.g., breakfast, snack). First a list was created with the foods reported in the Dutch national food consumption survey 2007–2010, sorted on their contribution to energy intake. The foods that contributed most to energy intake were included. It was then checked whether the obtained list covered the different food groups, the available variation in texture, taste and macronutrient composition, and different eating occasions.

The amount offered to the participants differed between foods, but in general they were smaller than commonly consumed servings. Together with a dietician it was decided on appropriate amounts, using standard portion sizes [22] as a starting point; the portions had to allow for multiple bites or sips, but not constrain further consumption. Finally, the participants were offered 12–65 g of solid foods, 75 g of semi-solid foods and 125 g of liquid foods. Furthermore, the foods were offered with cutlery where appropriate (e.g., yoghurt was offered with a spoon).

##### Reference foods

Three foods were selected to serve as a reference food and were offered twice to all participants. These were used to correct the eating rate data for the personal eating rate of the participants (see the part “Calculating food-specific eating rate and energy intake rate”).

The reference foods were apple (cultivar Elstar; 50 g), whole-wheat bread (AH Zaanse snijder volkoren heel, Albert Heijn BV.; 35 g (1 slice)) and semi-skimmed yoghurt (AH Milde yoghurt halfvol, Albert Heijn BV.; 75 g). These were selected because they cover different textures and are commonly consumed.

##### Participants

In total 89 healthy, normal weight, young adults (BMI 18.5–25 kg/m^2^, 18–30 years old) were recruited through posters, social media and e-mail; the e-mails were sent to a list of people interested in participating in studies at the Division of Human Nutrition (Wageningen University).

People who indicated to be interested in participation were invited to attend an information meeting. Here they received further information, and if they decided to participate they were asked to provide oral and written consent. Subsequently they were asked to have their height and weight measured and to fill out some questionnaires (including food allergies and intolerances, liking of foods under study, and problems with chewing or swallowing). People could not participate if they could not eat the reference foods because of allergies or intolerances (*n* = 3), did not like the reference foods (*n* = 1), or experienced problems with chewing or swallowing (*n* = 0).

Afterwards eligible participants were contacted and three test sessions were scheduled with each of the participants; additionally some participants (*n* = 32) were later asked to attend an extra test session, to fill in gaps that resulted from missing or unusable measurements. The final group of participants (*n* = 89) consisted of 69 females and 20 males, which were 21.2 ± 1.9 years old and had a BMI of 21.4 ± 1.9 kg/m^2^.

This study was approved by the medical ethical committee of Wageningen University (NL47315.081.13), and was conducted according to the guidelines laid down in the declaration of Helsinki.

##### Procedure

Test sessions were scheduled in November–December 2016 during lunchtime for every participant at a fixed time with approximately one week between test sessions. Participants were not assigned to a session during which foods were offered that they were not familiar with or disliked (i.e., a score below 3 on a five-point Likert scale; 1, Dislike–3, Neutral–5, Really like). Furthermore, participants were asked not to eat or drink anything other than water in the two hours before a test session. A test session took approximately 30 min.

During a test session participants (generally five) were seated in sensory booths with a computer screen in front of them. They first received some questions regarding their satiety level. They were asked when they last had something to eat or to drink (other than water), and to indicate on a ten-point Likert scale their level of hunger (1, Not hungry at all–10, Very hungry), level of fullness (1, Not full at all–10, Very full), and their desire to eat (1, Very weak–10, Very strong) [23].

Subsequently the participants were offered five foods. During their first and third session these included all three reference foods. The other foods were randomly assigned to a session. It, however, was made sure that the foods assigned to a single session offered variation in texture and taste. Furthermore, the order in which the foods were offered was randomized over participants.

Participants were instructed (both orally and in writing) to consume the foods like they normally would but without stops between bites or sips. Once they swallowed a bite or sip, they immediately had to take the next one. It was also allowed to take the next bite or sip before they had cleared their mouths. Furthermore, participants had to click on a button on the computer screen in front of them when taking the first bite or sip of a product. They could stop once they had finished the offered portion, or when two minutes had elapsed. When two minutes had elapsed (in 34% of the times a food was offered), a screen would appear with the instructions to stop after finishing the last bite or sip. In both cases, the participants had to again click on a button once they had swallowed the last bite or sip. The time between clicking the buttons was recorded, which represents the time spent eating. Afterwards participants were asked to indicate how much they liked the product on a nine-point Likert scale (1, Not tasty at all–9, Very tasty) and to neutralize their pallet with water and a cracker. One minute later they could request the next product.

After completing this procedure for all five products the participant were again asked to rate their level of hunger, level of fullness, and their desire eat on a ten-point Likert scale [23]. Finally, the participants were asked to report any comments (e.g., if they failed to follow instructions); Adherence to the instructions was not directly monitored. Furthermore, the amount eaten was measured by weighing the foods prior to and after consumption.

##### Calculating food-specific eating rate and energy intake rate

The observed eating rate (g/min) was determined by dividing the amount eaten (g) by the time spent eating (min). This number was then calibrated to correct for the personal eating rate of the participant; the observed eating rate was divided by a calibration factor based on how fast the participant ate the reference foods relative to the rest of the participants.

$$\text{Calibration}\;\text{factor}=\frac{\left(\frac{\mathrm{mean}\;\mathrm{ER}\;\mathrm{bread}\;(\mathrm{participant})}{\mathrm{mean}\;\mathrm{ER}\;\mathrm{bread}\;(\mathrm{group})})+(\frac{\mathrm{mean}\;\mathrm{ER}\;\mathrm{apple}\;(\mathrm{participant})}{\mathrm{mean}\;\mathrm{ER}\;\mathrm{apple}\;(\mathrm{group})})+(\frac{\mathrm{mean}\;\mathrm{ER}\;\mathrm{yoghurt}\;(\mathrm{participant})}{\mathrm{mean}\;\mathrm{ER}\;\mathrm{yoghurt}\;(\mathrm{group})}\right)}{3}$$

where: ER, eating rate. The eating rate of the tested foods was then determined by averaging the calibrated eating rates. Finally, energy intake rate (kJ/min) was obtained by multiplying eating rate (g/min) with the energy density (kJ/g) of the corresponding product.

#### 2.1.2. Additional Eating Rate Data

Data on the eating rate of 48 foods was derived from two other studies performed at Wageningen University to maximize the final dataset. In both studies normal-weight, young adults were instructed to eat as they normally would, but without stops between bites or sips. In one of the studies participants consumed one food per test session, and regardless of the food they had to finish 50 g [3]. The data from this study was calibrated, like in the current study, to correct for the personal eating rate of the participants. Calibration was based on how fast the participants ate two reference foods: i.e., semi-skimmed yoghurt and whole-wheat bread. In the other study participants consumed 6 products per test session (unpublished results). They received 50 g portions for semi-solids and solids, and 125 g portions for liquids. The data from this study did not need calibration as all products were consumed by all participants (*n* = 25).

### 2.2. Data on Other Food Properties

#### 2.2.1. Texture

All foods were assigned to a texture category using the definitions used by Stieger and van de Velde [19]:

- Liquids: Foods that flow and do not require chewing before swallowing (e.g., milk, beverages, yoghurt drinks);
- Semi-solids: Foods that are predominantly processed by squeezing them between tongue and palate, without the use of the molars (e.g., pudding, custard);
- Soft solids: Foods that require (initial) chewing between the molars, but do not have crispy sensations (e.g., cheese, processed meat);
- Hard solids: Crispy foods that require chewing between the molars and generally produce an acoustic sound emission during oral processing (e.g., crackers, raw vegetables, apples).

One researcher assigned the texture categories to all foods, and in case of doubt the categorization was discussed with a second researcher.

#### 2.2.2. Food Composition

Information on food composition (i.e., energy-, protein-, fat-, carbohydrate-, monosaccharide-, disaccharide-, polysaccharide-, fiber-, water- and sodium-content) was derived from the Dutch Food Composition Database. This database contains food composition data of foods commonly consumed in the Netherlands (Dutch Food Composition Database version 5.0, Dutch National Institute for Public Health and the Environment, Bilthoven, The Netherlands). In this database it is assumed that fiber contributes to the energy content of foods: i.e., 8 kJ/g.

#### 2.2.3. Taste

The taste intensities were retrieved from a study that used a trained sensory panel to score over 400 foods on sweet, sour, bitter, umami, salt and fat taste intensity using visual analogue scales [24,25,26]. Scores ranged from 0–100, with a higher score indicating a more intense taste.

#### 2.2.4. Food Groups

The foods were categorized into EPIC-Soft food groups (i.e., food groups developed for the European Prospective Investigation into Cancer and Nutrition (EPIC) study) to help describe the foods under study [27].

#### 2.2.5. Recommended Foods

The Netherlands Nutrition Centre uses the “Wheel of Five” to communicate the Dutch food-based recommendations to the public [28,29]. This Wheel of Five shows the type of foods needed to ensure the intake of the required nutrients. An online tool was used to see whether individual foods were part of the Wheel of Five (i.e., recommended foods), or not (i.e., not-recommend foods) [30].

### 2.3. Categorization of Foods According to Energy Intake Rate within Food Groups

The obtained dataset was manually inspected to identify groups of foods that distinguish themselves, based on their energy intake rate, from the other foods within the food group. First the foods were sorted on energy intake rate. The resulting list was then inspected to see whether certain foods tended to have higher or lower energy intake rate compared to the other foods within food group. These foods could be grouped based on any shared feature related to eating rate and/or energy density like, preparation/conservation method and food composition. For example, energy intake rate might differ between raw, boiled and fried foods; these preparation methods have the potential to affect both eating rate and energy density.

### 2.4. Statistical Analyses

SAS version 9.4 (SAS Institute Inc., Cary, NC, USA) was used for the statistical analyses. Means and standard deviations are given, unless stated otherwise. *p* values of <0.05 were considered statistically significant.

To validate the calibration factor analyses of variance was used to check whether the eating rate of the participants divided by the group mean was the same between the reference foods. With paired samples *t*-tests it was investigated whether there was a differences in eating rate the first and second time the participants were offered the reference foods, and whether the satiety scores were different before and after the test sessions. Pearson correlation analysis was performed to see if eating rate was correlated with liking.

Secondly, the associations between eating rate and energy intake rate and food properties were investigated both in the whole dataset and in the dataset after excluding liquids. Quartiles were created for both eating rate and energy intake rate. Linear regression analyses were performed to investigate whether there was a linear trend between the eating rate and energy intake rate quartiles, and food properties (i.e., eating rate, energy intake rate, food composition and taste intensities). Chi-square tests were performed to see whether food properties (i.e., texture, food groups and recommended foods) were distributed differently over the eating rate and energy intake rate quartiles. Furthermore, Pearson correlation analysis was used to assess the correlation between eating rate and energy intake rate and food properties: i.e., food composition and taste intensities. These correlation analyses were repeated after excluding the eating rate data from previous studies. Non-parametric tests were performed to investigate whether eating rate and energy intake rate differed between the texture groups. The Kruskal-Wallis test was used to see if the median was significantly different between texture groups, and the Jonckheere-Terpstra test was used to see whether there was a linear trend. Moreover, with the use of independent samples *t*-test it was investigated whether eating rate and energy intake rate differed between recommended and not-recommended foods.

## 3. Results

### 3.1. Descriptives

**Figure 1:**
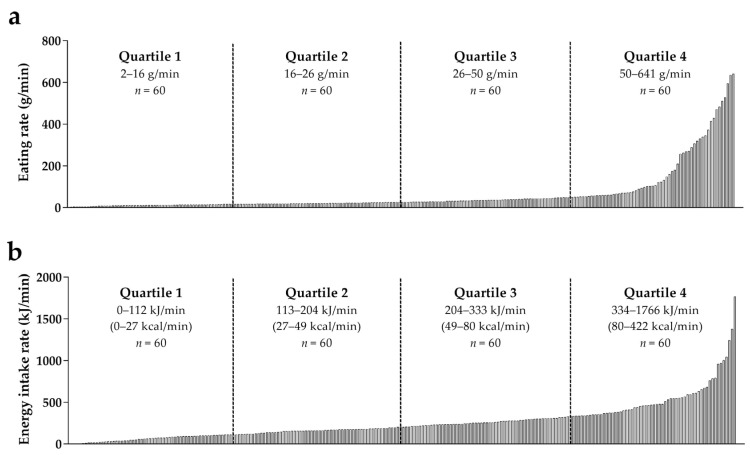
Food-specific eating rate (**a**) and food-specific energy intake rate (**b**) of the foods in the dataset (*n* = 240).

The final dataset consisted of 240 foods; 192 from the current study, 37 from the study by [3], and 11 from the unpublished study (Figure 1 and Table S1). The dataset covers a wide variety of foods (Tables S2 and S3). The eating rate of the included foods ranged from 2 g/min (i.e., rice waffle) to 641 g/min (i.e., apple juice), and their energy intake rate ranged from 0 kJ/min (0 kcal/min) (i.e., water) to 1766 kJ/min (422 kcal/min) (i.e., full fat, chocolate-flavored milk).

Figure 1a displays the eating rate quartiles of the foods in the dataset. The food groups “Cereals and cereal products”, “Sugar and confectionary” and “Cakes” were predominantly present in the first eating rate quartile, while “Non-alcoholic beverages”, “Dairy products”, and “Soups, bouillon” were predominantly present in the fourth eating rate quartile (Table S2).

Figure 1b displays the energy intake rate quartiles. The food groups “Vegetables” and “Soups, bouillon” were predominantly present in the first quartile, while “Dairy products”, “Cakes” and “Snacks” were predominantly present in the higher quartiles (Table S2). Furthermore, within the “Non-alcoholic beverages” there was a clear divide; all non- and very low-caloric beverages were present in the first energy intake rate quartile, while the rest of the beverages were predominantly present in the fourth energy intake rate quartile.

### 3.2. Data Checks

The eating rate of the reference foods did not differ significantly between the first and second time it was offered (paired samples *t*-tests: for apple *p* = 0.07, for bread *p* = 0.62 and for yoghurt *p* = 0.15). The mean eating rate for apple was 36 ± 11 g/min at session 1 and 38 ± 11 g/min at session 3, for bread 10 ± 4.2 g/min at session 1 and 10 ± 4.3 g/min at session 3, and for yoghurt 98 ± 32 g/min at session 1 and 103 ± 30 g/min at session 3. The coefficient of variation (i.e., standard deviation/mean × 100) at first consumption was 31% for apple, 42% for bread, and 33% for yoghurt. Furthermore, the eating rate of the participants divided by the group average was the same within the three reference foods (*F*(2, 176) = 0.06, *p* = 0.95).

The liking scores were not correlated with the eating rate of apple, but the liking scores were associated with the eating rate of bread and yoghurt (apple *r* = 0.13, *p* =0.09; bread *r* = 0.27, *p* ≤ 0.001; yoghurt *r* = 0.27, *p* ≤ 0.0001). For bread the average eating rate was 9 g/min when it was not liked (i.e., liking score ≤4) and 11 g/min when it was liked (i.e., liking score ≥5). For yoghurt it was 84 g/min when it was not liked and 103 g/min when it was liked.

The participants were less hungry, felt more full and their desire to eat was less at the end of the test sessions compared to the start of the test sessions (paired samples *t*-test: for all three satiety scores *p* < 0.0001). The average scores at the end of the test sessions were 3.8 for level of hunger, 6.4 level of fullness, and 4.5 for desire to eat (on 10-point Likert scales, anchored from “not at all” to “very”).

### 3.3. Eating Rate and Other Food Properties

#### 3.3.1. Food Composition

Several associations between eating rate and food composition were found (Table 1). Eating rate was negatively correlated with energy density (*r* = −0.45, *p* < 0.0001), macronutrient content (protein content *r* = −0.31, *p* < 0.0001; fat content *r* = −0.29, *p* < 0.0001; carbohydrate content *r* = −0.33, *p* < 0.0001). The same was true for fiber content (*r* = −0.33, *p* < 0.0001) and sodium content (*r* = −0.31, *p <* 0.0001). Water content was positively correlated with eating rate (*r* = 0.46, *p* < 0.0001). Mono- and disaccharide content was not correlated with eating rate.

After excluding liquids from the dataset some differences were found in the associations between eating rate and food properties (Table 1). The association between water content and eating rate became stronger (before *r* = 0.46, *p* < 0.0001; after *r* = 0.61, *p* < 0.0001). Similarly, the correlation between eating rate and energy density became more pronounced (before *r* = −0.45, *p* < 0.0001; after *r* = −0.57, *p* < 0.0001).

Excluding the data from previous did not change the results.

#### 3.3.2. Taste Intensity

Some associations between eating rate and taste intensity were found (Table 1). Sweet and bitter taste intensity were not correlated with eating rate. Salt, fat and umami taste intensity were negatively correlated with eating rate (salt *r* = −0.27, *p* < 0.001; fat *r* = −0.21, *p* < 0.01; umami *r* = −0.17, *p* = 0.01), while sour taste intensity was positively correlated with eating rate (*r* = 0.34, *p* < 0.0001).

After excluding liquids from the dataset, the correlations between eating rate and sour taste intensity became stronger (before *r* = 0.34, *p* < 0.0001; after *r* = 0.48, *p* < 0.0001), while the correlations between eating rate and umami taste intensity (before *r* = −0.17, *p* = 0.01; after *r* = −0.00, *p* > 0.99) and fat taste intensity (before *r* = −0.21, *p* < 0.01; after *r* = −0.01, *p* = 0.84) disappeared. Finally, excluding the data from previous studies did not change the results.

#### 3.3.3. Texture

The texture groups (i.e., liquids, semi-solids, soft solids and hard solid) were not equally distributed over the eating rate quartiles (Chi-square: *p* < 0.0001) (Table 2). The hard solids were mainly present in the lower quartiles, while the semi-solids and liquids were mainly present in the upper quartiles. Moreover, the mean eating rate was 306 ± 177 g/min for liquids, 63 ± 40 g/min for semi-solids, 30 ± 16 g/min for soft solids and 19 ± 15 for hard solids. The median eating rate decreased as the food texture became more solid and harder (Kruskal-Wallis test: *H*(3) = 111.85, *p* < 0.0001); Jonckheere-Terpstra test: *J* = 7954, *z* = −10.98, *p* < 0.0001).

#### 3.3.4. Dutch Recommendations

The eating rate of the recommended foods (64 ± 97 g/min) was not significantly different from that of the not recommended foods (65 ± 119 g/min) (independent samples *t*-test: *t*(238) = 0.09, *p* = 0.93). After excluding liquids from the dataset the eating rate of the recommended foods (35 ± 23 g/min) remained not significantly different from that of the not recommended foods (28 ± 25 g/min) (independent samples *t*-test: *t*(208) = −1.85, *p* = 0.07).

### 3.4. Energy Intake Rate And Other Food Properties

#### 3.4.1. Food Composition

Energy intake rate was positively associated with fat content (*r* = 0.16, *p* = 0.01) (Table 1). Fiber content was negatively correlated with energy intake rate (*r* = −0.20, *p* < 0.01). After excluding liquids from the dataset an association between energy intake rate and energy density became apparent (before *r* = 0.08, *p* = 0.19; after *r* = 0.43, *p* < 0.0001). Similarly, an association between water content and energy intake rate became apparent (before *r* = −0.03, *p* = 0.63; after *r* = −0.33, *p* < 0.0001).

#### 3.4.2. Taste Intensity

Table 1 shows that energy intake rate was positively associated with fat, sweet and sour taste intensity (fat taste intensity *r* = 0.28, *p* < 0.0001; sweet taste intensity *r* = 0.35, *p* < 0.0001; sour taste intensity *r* = 0.15, *p* = 0.03). After excluding liquids the association with sour taste intensity disappeared and a positive association with salt taste intensity became apparent (sour taste intensity *r* = −0.10, *p* = 0.18; salt taste intensity *r* = 0.19, *p* < 0.01).

#### 3.4.3. Texture

The texture groups were not equally distributed over the energy intake rate quartiles (Chi square, *p* < 0.0001) (Table 2). Liquids were predominantly present in the first and fourth quartile. Energy intake rate, however, was not significantly different between the texture groups (Kruskal-Wallis test: *H*(3) = 6.21, *p* = 0.10).

#### 3.4.4. Dutch Recommendations

The energy intake rate of the recommended foods (147 ± 137 kJ/min (35 ± 33 kcal/min)) was significantly lower than that of the not recommended foods (312 ± 250 kJ/min (75 ± 60 kcal/min)) (independent samples *t*-test: *t*(228.04) = 6.61, *p* < 0.0001). After excluding liquids from the dataset the energy intake rate of the recommended foods (133 ± 97 kJ/min (32 ± 23 kcal/min)) remained significantly lower than that of the not recommended foods (269 ± 145 kJ/min (64 ± 35 kcal/min)) (independent samples *t*-test: *t*(180.63) = 7.98, *p* < 0.0001).

### 3.5. Energy Intake Rate of Foods within Food Groups

Table 3 shows the categorization of foods, according to their energy intake rate (kJ/min), within the food groups (see Table S4 for the same table with energy intake rate expressed in kcal/min). For several food groups this categorization was based on differences in both eating rate and energy density. For example, in the “Potatoes” food group, mashed potatoes had a relatively high energy intake rate as the result of a high eating rate (52 g/min), while for fried potatoes and French fries this was the result of a high energy density (1107 kJ/100 g (265 kcal/100 g)). Similarly, in the “Dairy products” food group, both cheese and plain yoghurt and fromage frais had a relatively low energy intake rate. For the cheeses this was the result of a low eating rate (19 g/min), and for the plain yoghurt and fromage frais this was the result of a low energy density (215 kJ/100 g (51 kcal/min)).

For other food groups categorization was predominantly based on differences in eating rate (e.g., “Cereals and cereal products” and “Sugar and confectionery”), and for other food groups categorization was predominantly based on differences in energy density (e.g., “Non-alcoholic beverages”).

## 4. Discussion

The aim of the current study was to assess the eating rate and energy intake rate of the foods commonly consumed in the Netherlands, to map the characteristics of slow and fast foods, and to explore the opportunities for a diet with a low energy intake rate. Food-specific eating rate was obtained for 240 foods. Eating rate ranged from 2–641 g/min, and energy intake rate ranged from 0–1766 kJ/min (0–422 kcal/min). After excluding liquids these ranges were considerably smaller (i.e., 2–147 g/min and 10–761 kJ/min (2–182 kcal/min)), as the liquids were consumed more quickly than the semi-solids and solids. Besides texture also food composition was associated eating rate. Eating rate was inversely associated with energy density and fiber content, and positively associated with water content. No clear association was found between eating rate and taste intensity. Moreover, within the food groups we were able to identify groups of food that distinguished themselves from the other foods in the food group based on their energy intake rate. Hereby demonstrating that natural variation in energy intake rate is present in the Dutch diet.

This is the first study to report the eating rate and energy intake rate of a large number of foods that represent a whole diet (i.e., the Dutch diet). In line with previous studies a large variation in eating rate was found, with the liquids and semi-solids being responsible for most variation [2,3]. Eating rate was lower when the texture was more solid and harder. To illustrate, water had a high eating rate (i.e., 339 g/min), while wholemeal crispbread had a low eating rate (i.e., 5 g/min). Previous studies have already shown that food texture directly affects eating rate [13,14]. As Hutchings and Lillford [31] show in their model the time to process a food depends on the degree of structure and lubrication. Foods that require more chewing and lubrication will take more time to process and therefore will have a lower eating rate. This is supported by the finding that water content was positively associated with eating rate.

Regarding food composition, water content was the best predictor of eating rate, which is in line with previous research [2,3]. On the other hand, energy density, and protein, fat, carbohydrate and fiber content were inversely associated with eating rate. The correlations between eating rate and energy density, water content, and carbohydrate content became stronger after excluding liquids, indicating that the association between eating rate and food composition is different in liquids compared to non-liquids. Energy density, fiber and water content all are associated with the texture of a food, which could explain the association with eating rate [19]. For fat, however, the relation with eating rate might be more complex. On the one hand it contributes to the energy density of foods, which is negatively associated with eating rate. On the other hand fat can act as a lubricator, which would have an inverse effect [19,31]. Further research is needed to better understand the relation between fat content and the eating rate of commonly consumed foods.

For the relation between taste intensity and eating rate we expected to find a negative association. Some studies have shown that taste intensity is inversely associated with eating rate, but this is not consistently found [2,14,20]. In the current study salt taste intensity was inversely correlated with eating rate, also after excluding liquids. The same was true for sodium content. This can partly be explained by the presence of foods from the “Cereals and cereal products” food group (e.g., salty biscuits) in the lower eating rate quartiles. Furthermore, sour taste intensity was positively correlated with eating rate, also after excluding liquids. This correlation can be explained by the presence of foods from the “Dairy products” and “Fruits, nuts and seeds” food group in the higher eating rate quartiles (after excluding liquids). No associations were found between eating rate and sweet and bitter taste intensity. This could be due to the omnipresence of sweet and the lack of bitter foods in our diet [32]. Across eating rate quartiles sweet taste intensity and mono- and disaccharide content remained relatively high, while bitter taste intensity remained very low.

The current study was designed to measure food-specific eating rate; other factors influencing eating rate were standardized as much as possible. Eating rate was measured in a laboratory setting and participants were not allowed to take breaks in between bites or sips. Moreover, a calibration factor was used to correct the data for the personal eating rate of the participants, as the participants did not consume all foods. This calibration factor assumes that eating rate is a personal characteristic [33,34]. The fact that the eating rate of the participants compared to the group mean was similar within products confirms this assumption and validates the use of the calibration factor. The absolute numbers, however, will still depend on the population, but the relative differences in eating rate between foods are expected to be similar. Moreover, participants were not offered foods if they indicated beforehand that they did not like it. There, however, was a significant positive correlation between the liking scores and eating rate for bread and yoghurt. Perhaps this is because these products usually are not consumed plain. Nonetheless this is not expected to have altered our findings, considering the small absolute differences in eating rate when the product is liked and when it is not liked.

Furthermore, it was chosen not to offer equal portions for all foods, like previous studies with a similar design have offered 50 g portions [2,3,17]. This was not feasible considering the wide range of foods included in this study. It, for example, would mean that participants had to eat a complete roll of peppermint (i.e., approximately 50 g). On the other hand, offering portions smaller than 50 g would not be informative for other foods (e.g., liquids). Therefore it was decided to offer portions that allowed for multiple bites/sips, but did not constrain further consumption. This is reflected in the satiety scores; although the participants felt fuller at the end of the test sessions they indicated they could still eat more. Furthermore, data was added from previous studies that used slightly different methods (e.g., regarding the portions offered), but this did not affect the results. Excluding data from these previous studies did not change the results.

The results of the current study provide valuable information on the eating rate and energy intake rate of commonly consumed foods. They improve our understanding of the determinants of eating rate and energy intake rate, although the number of repetitions per food is limited. Furthermore, the dataset is not complete but the foods included were carefully selected to represent the range of foods present in the Dutch diet. The eating rate of foods, however, might be different when not consumed in isolation. Nonetheless, when a meal component is replaced by an alternative with a lower eating rate this is expected to result in a lower overall eating rate, as illustrated by Bolhuis et al. [1]. They, for example, showed that a hamburger was eaten more slowly when the soft bread was replaced by hard bread, which has a lower eating rate. Moreover, this example shows that lowering the eating rate—and therefore the energy intake rate—of a diet does not necessarily require big adaptations.

The current study shows that it is possible to choose alternatives with a lower energy intake rate across the diet. This is also expected to be true for other western countries because of the similarities between western diets [27,35]. Alternatives with a lower energy density can be chosen from either another food group or from the same food group. Differences in energy intake rate within a food group were, in general, either the result of a difference in eating rate or a difference in energy density; this reflects the negative association between eating rate and energy density. Furthermore, the results show that adhering to the current dietary guidelines will lower energy intake rate in most individuals [29,30,36]. Most individuals do not consume enough fruit and vegetables, while these have a low energy intake rate [37]. Moreover, the energy intake rate of not-recommended foods was twice as high compared to the recommended foods. This suggests that there is room for improvement regarding the energy intake rate of the diet for people that are already adhering to the guidelines, but especially for people that are not. When not just considering the commonly consumed foods even bigger differences could be obtained; for example with foods designed to lower eating rate, and therefore energy intake rate [38]. Selecting foods with a low energy intake rate will make it easier for people to control their energy intake because of the satiating capacity of these foods [1,11,13].

## 5. Conclusions

The foods present in the Dutch diet vary greatly in eating rate and energy intake rate. Foods with a low eating rate are mainly characterized by a solid texture, high energy density and low water content. Foods with a low energy intake rate are by definition characterized by a low eating rate and low energy density. Furthermore, we have demonstrated that it is possible to choose alternatives with a lower energy intake rate, either from the same or another food group. This study, therefore, demonstrates that commonly consumed foods provide opportunities for reducing energy intake rate, and may serve as a starting point when designing an intervention to reduce energy intake by selecting foods with a low energy intake rate (kJ/min). Such an intervention, targeting both the eating rate and energy density of foods, is expected to be more effective compared to an intervention that only targets the eating rate or the energy density of foods.

## Figures and Tables

**Table 1:** Pearson correlations between food-specific eating rate and energy intake rate, and food properties; with and without considering liquids.

	Eating Rate (g/min)				Energy Intake Rate (kJ/min)			
	Including Liquids ^1^		Excluding Liquids ^2^		Including Liquids ^1^		Excluding Liquids ^2^	
	*r*	*p*	*r*	*p*	*r*	*p*	*r*	*p*
Food Composition								
Energy (kJ/100 g)	−0.45	<0.0001	−0.57	<0.0001	0.08	0.19	0.43	<0.0001
Protein (g/100 g)	−0.31	<0.0001	−0.27	<0.0001	−0.002	0.97	0.19	<0.01
Fat (g/100 g)	−0.29	<0.0001	−0.31	<0.0001	0.16	0.01	0.47	<0.0001
Carbohydrate (g/100 g)	−0.33	<0.0001	−0.48	<0.0001	−0.02	0.75	0.12	0.07
Mono- and disaccharides (g/100 g)	−0.05	0.47	0.01	0.89	0.001	0.98	0.02	0.78
Polysaccharides (g/100 g)	−0.34	<0.0001	−0.47	<0.0001	−0.13	<0.05	−0.02	0.82
Fiber (g/100 g)	−0.33	<0.0001	−0.33	<0.0001	−0.20	<0.01	−0.16	0.02
Water (g/100 g)	0.46	<0.0001	0.61	<0.0001	−0.03	0.63	−0.33	<0.0001
Sodium (mg/100 g)	−00.31	<0.0001	−0.30	<0.0001	−0.07	0.25	0.11	0.10
Taste Intensities								
Sweet	0.11	0.09	−0.02	0.83	0.35	<0.0001	0.41	<0.0001
Sour	0.34	<0.0001	0.48	<0.0001	0.15	0.03	−0.10	0.18
Bitter	0.08	0.21	−0.01	0.92	−0.06	0.38	−0.08	0.26
Umami	−0.17	0.01	−0.00	>0.99	−0.12	0.08	0.05	0.47
Salt	−0.27	<0.001	−0.19	<0.01	−0.06	0.39	0.19	<0.01
Fat	−0.21	<0.01	−0.01	0.84	0.28	<0.0001	0.61	<0.0001

^1^ *n* = 240 for correlations with food composition variables, and *n* = 224 for correlations with taste intensities; ^2^ *n* = 210 for correlations with food composition variables, and *n* = 194 for correlations with taste intensities.

**Table 2:** Frequency (*n* (%)) of texture groups and recommended foods in the eating rate and energy intake rate quartiles (*n* = 240).

	Eating Rate (g/min)				*p* ^2^	Energy Intake Rate (kJ/min) ^1^				*p* ^2^
	Quartile 1	Quartile 2	Quartile 3	Quartile 4		Quartile 1	Quartile 2	Quartile 3	Quartile 4	
	2–16 g/min (*n* = 60)	16–26 g/min (*n* = 60)	26–50 g/min (*n* = 60)	50–641 g/min (*n* = 60)		0–112 kJ/min (*n* = 60)	113–204 kJ/min (*n* = 60)	204–333 kJ/min (*n* = 60)	334–1766 kJ/min (*n* = 60)	
Food Texture					<0.0001					0.0001
Liquids	0 (0.0)	0 (0.0)	1 (1.7)	29 (48.3)		10 (16.7)	2 (3.3)	0 (0.0)	18 (30.0)	
Semi-solids	3 (5.0)	3 (5.0)	6 (10.0)	15 (25.0)		5 (8.3)	8 (13.3)	7 (11.7)	7 (11.7)	
Soft solids	18 (30.0)	37 (61.7)	40 (66.7)	12 (20.0)		24 (40.0)	29 (48.3)	34 (56.7)	20 (33.3)	
Hard solids	39 (65.0)	20 (33.3)	13 (21.7)	4 (6.7)		21 (35.0)	21 (35.0)	19 (31.7)	15 (25.0)	
Dutch Dietary Guidelines					>0.05					<0.0001
Recommended	14 (23.3)	14 (23.3)	26 (43.3)	20 (33.3)		35 (58.3)	21 (35.0)	12 (20.0)	6 (10.0)	
Not recommended	46 (76.7)	46 (76.7)	34 (56.7)	40 (66.7)		25 (41.7)	39 (65.0)	48 (80.0)	54 (90.0)	

^1^ Energy intake rate quartiles (kcal/min); Quartile 1= 0–27 kcal/min, Quartile 2 = 27–49 kcal/min, Quartile 3 = 49–80 kcal/min, Quartile 4 = 80–422 kcal/min; ^2^ *p*-value Chi-square test.

**Table 3:** Energy intake rate (kJ/min) of foods relative to the other foods within the food group ^1^.

Food Group	Energy Intake Rate (kJ/min) Relative to Food Group		
	Low	Medium	High
Potatoes (*n* = 6)			
Description	Boiled potatoes ^2^ (*n* = 2)		Mashed and (deep-)fried potatoes (*n* = 4)
Energy intake rate	76 (64–87) kJ/min		248 (183–308) kJ/min
Eating rate	23 (18–28) g/min		32 (22–52) g/min
Energy density	332 (311–352) kJ/100 g		917 (349–1300) kJ/100 g
Vegetables (*n* = 24)			
Description	Raw vegetables ^2^ (*n* = 5)	Boiled ^2^ and pickled vegetables (*n* = 17)	Vegetables with added energy (*n* = 2)
Energy intake rate	28 (10–73) kJ/min	46 (10–119) kJ/min	131 (108–153) kJ/min
Eating rate	36 (12–76) g/min	37 (13–89) g/min	48 (44–51) g/min
Energy density	81 (52–139) kJ/100 g	121 (70–291) kJ/100 g	275 (247–303) kJ/100 g
Legumes (*n* = 2)			
Description	Tinned brown beans ^2^ (*n* = 1)		Tinned beans in tomato sauce (*n* = 1)
Energy intake rate	129 kJ/min		176 kJ/min
Eating rate	28 g/min		45 g/min
Energy density	460 kJ/100 g		393 kJ/100 g
Fruits, nuts and olives (*n* = 20)			
Description	Fruit (excluding soft fruit) ^2^ (*n* = 8)	Olives, conserved fruit and soft fruit ^2^ (*n* = 7)	Nuts ^3^, apple sauce (*n* = 5)
Energy intake rate	111 (60–176) kJ/min	164 (99–278) kJ/min	349 (206–479) kJ/min
Eating rate	46 (26–73) g/min	52 (12–97) g/min	39 (8–147) g/min
Energy density	243 (193–331) kJ/100 g	487 (123–1382) kJ/100 g	2053 (325–2586) kJ/100 g
Dairy products (*n* = 26)			
Description	Plain yoghurt and fromage frais ^3^, cheese ^2^ (*n* = 8)	Deserts other than plain yoghurt or fromage frais (*n* = 8)	Dairy drinks ^3^ (*n* = 10)
Energy intake rate	225 (146–319) kJ/min	412 (231–546) kJ/min	749 (200–1766) kJ/min
Eating rate	58 (12–132) g/min	80 (33–122) g/min	322 (71–527) g/min
Energy density	776 (156–1529) kJ/100 g	643 (300–1453) kJ/100 g	232 (122–375) kJ/100 g
Cereals and cereal products (*n* = 56)			
Description	Hard and dry products ^3^, plain bread slices ^3^ (*n* = 23)		Other (e.g., bread with topping, buns, pasta, rice) ^3^ (*n* = 33)
Energy intake rate	137 (37–258) kJ/min		241 (106–549) kJ/min
Eating rate	9 (2–13) g/min		24 (10–54) g/min
Energy density	1639 (990–2261) kJ/100 g		1069 (555–1481) kJ/100 g
Meat and meat products (*n* = 18)			
Description	Fresh meat (excluding minced meat) ^3^ (*n* = 2)		Minced meat ^3^ and processed meat (*n* = 16)
Energy intake rate	176 (117–234) kJ/min		300 (71–654) kJ/min
Eating rate	27 (18–35) g/min		29 (13–58) g/min
Energy density	664 (661–667) kJ/100 g		1039 (520–1804) kJ/100 g
Fish and shellfish (*n* = 6)			
Description		Fish and fish products ^2^ (*n* = 6)	
Energy intake rate		234 (123–372) kJ/min	
Eating rate		31 (24–48) g/min	
Energy density		761 (414–918) kJ/100 g	
Eggs and egg products (*n* = 1)			
Description		Boiled egg ^2^ (*n* = 1)	
Energy intake rate		173 kJ/min	
Eating rate		32 g/min	
Energy density		535 kJ/100 g	
Sugar and confectionery (*n* = 19)			
Description	Hard confectionary (non-chocolate), ice cream (*n* = 5)	Soft confectionary (non-chocolate) (*n* = 6)	Chocolate, candy bars, fruit drink (*n* = 8)
Energy intake rate	100 (63–156) kJ/min	210 (140–278) kJ/min	369 (185–610) kJ/min
Eating rate	9 (4–16) g/min	14 (9–19) g/min	47 (8–268) g/min
Energy density	1357 (856–1676) kJ/100 g	1513 (1358–1796) kJ/100 g	1898 (227–2342) kJ/100 g
Cakes (*n* = 26)			
Description	Dry cakes, biscuits (*n* = 15)		Cakes, pies, pastries, puddings (non-milk-based) (*n* = 11)
Energy intake rate	317 (166–685) kJ/min		436 (239–636) kJ/min
Eating rate	17 (9–35) g/min		33 (18–58) g/min
Energy density	1861 (1314–2205) kJ/100 g		1409 (826–1868) kJ/100 g
Non-alcoholic beverages (*n* = 15)			
Description	Non- and very low caloric beverages ^3^ (*n* = 5)		Caloric beverages (*n* = 10)
Energy intake rate	1 (0–3) kJ/min		673 (92–1379) kJ/min
Eating rate	334 (56–635) g/min		365 (59–641) g/min
Energy density	1 (0–5) kJ/100 g		173 (68–232) kJ/100 g
Alcoholic beverages (*n* = 1)			
Description		Pilsner beer (*n* = 1)	
Energy intake rate		198 kJ/min	
Eating rate		106 g/min	
Energy density		187 kJ/100 g	
Condiments and sauces (*n* = 7)			
Description	Tomato sauces (*n* = 3)		Mayonnaises and similar (*n* = 4)
Energy intake rate	93 (78–111) kJ/min		262 (207–321) kJ/min
Eating rate	28 (17–44) g/min		20 (11–31) g/min
Energy density	375 (253–546) kJ/100 g		1493 (975–2733) kJ/100 g
Soups, bouillon (*n* = 7)			
Description	Soup from cube or package (*n* = 3)		Soup with more (semi-) solid components (*n* = 4)
Energy intake rate	50 (38–74) kJ/min		137 (104–199) kJ/min
Eating rate	89 (41–174) g/min		66 (59–70) g/min
Energy density	86 (22–140) kJ/100 g		214 (148–337) kJ/100 g
Snacks (*n* = 6)			
Description	Spring roll fried (*n* = 1)		Other warm savory snacks (*n* = 5)
Energy intake rate	204 kJ/min		494 (401–761) kJ/min
Eating rate	27 g/min		36 (27–51) g/min
Energy density	757 kJ/100 g		1366 (1139–1596) kJ/100 g

^1^ Table S4 shows the same table with energy intake rate expressed in kcal/min and energy density in kcal/100g; ^2^ recommended foods; ^3^ both recommended and not recommended food.

## Supplementary Materials

The following are available online at www.mdpi.com/2304-8158/6/10/87/s1, Table S1: A detailed description of the foods included in the dataset (*n* = 240), Table S2: Frequency (*n* (%)) of food groups in the eating rate and energy intake rate quartiles (*n* = 240), Table S3: Characteristics (mean ± SD) of the foods in the eating rate and energy intake rate quartiles (*n* = 240): eating rate, energy intake rate, food composition and taste, Table S4: Energy intake rate (kcal/min) of foods relative to the other foods within the food group.
