# Supplementary material for: The Availability of Slow and Fast Calories in the Dutch Diet: The Current Situation and Opportunities for Interventions
Source: Foods. 2017 Oct 2;6(10):87. doi: 10.3390/foods6100087 (PMC5664026; doi:10.3390/foods6100087)
Supplement: Supplementary file 1 [file foods-06-00087-s001.pdf]

Supplementary file

The availability of slow and fast calories in the Dutch diet: The current situation and opportunities for interventions

Janet van den Boer, Melanie Werts, Els Siebelink, Cees de Graaf, Monica Mars

Table S1 A detailed description of the foods included in the dataset (*n* = 240).

Food	Food Group	Texture	Source eating rate data	Portion size (g)	*n*	Energy intake rate		Eating rate	Energy density	
						kJ/min	kcal/min	(g/min)	kJ/100g	kcal/100g
Mineral water (Spa)	Non-alcoholic beverages	Liquid	Current study	125	5	0 ± 0	0 ± 0	210 ± 53	0	0
Water	Non-alcoholic beverages	Liquid	Current study	125	5	0 ± 0	0 ± 0	339 ± 219	0	0
Tea	Non-alcoholic beverages	Liquid	Previous study	50	2	0 ± 0	0 ± 0	428 ± 165	0	0
Cola light soft drink	Non-alcoholic beverages	Liquid	Previous study	50	4	0 ± 0	0 ± 0	635 ± 508	0	0
Coffee	Non-alcoholic beverages	Liquid	Current study	125	5	3 ± 1	1 ± 0	56 ± 14	5	1
Bean sprouts boiled	Vegetables	Hard-solid	Current study	50	5	10 ± 3	2 ± 1	13 ± 4	75	18
Lettuce iceberg raw	Vegetables	Hard-solid	Previous study	50	3	10 ± 6	2 ± 1	17 ± 9	62	15
Chicory boiled	Vegetables	Soft-solid	Current study	50	5	15 ± 7	4 ± 2	21 ± 10	70	17
Carrot raw	Vegetables	Hard-solid	Previous study	50	2	17 ± 10	4 ± 2	12 ± 7	139	33
Cucumber with skin raw	Vegetables	Hard-solid	Previous study	50	4	18 ± 9	4 ± 2	34 ± 17	54	13
Leek boiled	Vegetables	Soft-solid	Current study	50	4	21 ± 10	5 ± 2	23 ± 11	91	22
Cucumber without skin raw	Vegetables	Hard-solid	Current study	50	5	22 ± 7	5 ± 2	43 ± 13	52	12
Silver-skin onion sweet pickled	Vegetables	Hard-solid	Current study	25	5	25 ± 5	6 ± 1	17 ± 3	142	34
Cabbage oxheart boiled	Vegetables	Soft-solid	Current study	50	5	27 ± 10	7 ± 2	31 ± 11	88	21
Beans French boiled	Vegetables	Soft-solid	Current study	50	5	31 ± 13	7 ± 3	29 ± 12	105	25
Courgettes boiled	Vegetables	Soft-solid	Current study	50	5	31 ± 8	7 ± 2	39 ± 10	79	19
Mushroom boiled	Vegetables	Soft-solid	Current study	50	4	33 ± 8	8 ± 2	36 ± 9	90	21
Spinach frozen boiled	Vegetables	Semi-solid	Current study	50	5	37 ± 30	9 ± 7	35 ± 28	104	25
Rice waffle	Cereals and cereal products	Hard-solid	Previous study	50	2	37 ± 38	9 ± 9	2 ± 2	1587	374
Stock from cube	Soups, bouillon	Liquid	Current study	75	4	38 ± 13	9 ± 3	174 ± 59	22	5
Soup vegetable based dried packet	Soups, bouillon	Liquid	Current study	75	5	39 ± 11	9 ± 3	41 ± 12	95	23
Celeriac boiled	Vegetables	Soft-solid	Current study	50	5	40 ± 20	9 ± 5	23 ± 11	175	42

Broccoli boiled	Vegetables	Soft-solid	Current study	50	5	47 ± 21	11 ± 5	42 ± 19	112	27
Sweet pepper red boiled	Vegetables	Soft-solid	Current study	50	5	48 ± 16	11 ± 4	42 ± 14	113	27
Gherkins sweet pickled	Vegetables	Hard-solid	Current study	50	5	49 ± 14	12 ± 3	43 ± 13	113	27
Cauliflower boiled	Vegetables	Soft-solid	Current study	50	5	58 ± 23	14 ± 6	61 ± 24	95	23
Pear with skin	Fruits, nuts and olives	Soft-solid	Current study	50	5	60 ± 15	14 ± 4	26 ± 7	231	55
Peppermint	Sugar and confectionery	Hard-solid	Current study	20	5	63 ± 44	15 ± 11	4 ± 3	1676	394
Potatoes without skins boiled	Potatoes and other tubers	Soft-solid	Previous study	50	2	64 ± 24	15 ± 6	18 ± 7	352	83
Boiled sweets	Sugar and confectionery	Hard-solid	Current study	17	5	65 ± 62	16 ± 15	4 ± 4	1615	380
Ham lean boiled	Meat and meat products	Soft-solid	Previous study	50	22	71 ± 32	17 ± 8	13 ± 6	564	135
Tomato raw	Vegetables	Hard-solid	Previous study	50	3	73 ± 19	17 ± 5	76 ± 20	96	23
Soup portion	Soups, bouillon	Liquid	Current study	75	5	74 ± 13	18 ± 3	53 ± 9	140	33
Crispbread wholemeal	Cereals and cereal products	Hard-solid	Current study	13	5	75 ± 15	18 ± 4	5 ± 1	1529	363
Brussels sprouts boiled	Vegetables	Soft-solid	Current study	50	5	76 ± 14	18 ± 3	40 ± 7	189	45
Ketchup tomato	Condiments and sauces	Semi-solid	Current study	25	4	78 ± 72	19 ± 17	24 ± 22	327	77
Bread brown wheat	Cereals and cereal products	Soft-solid	Previous study	50	25	83 ± 25	20 ± 6	8 ± 3	1000	236
Crispbakes Dutch wholemeal	Cereals and cereal products	Hard-solid	Current study	13	5	87 ± 22	21 ± 5	5 ± 1	1664	394
Potatoes boiled with skin	Potatoes and other tubers	Soft-solid	Current study	50	5	87 ± 30	21 ± 7	28 ± 10	311	74
Apple without skin	Fruits, nuts and olives	Hard-solid	Previous study	50	25	89 ± 25	21 ± 6	36 ± 10	247	58
Breadsticks	Cereals and cereal products	Hard-solid	Current study	13	5	89 ± 17	21 ± 4	5 ± 1	1694	401
Ketchup curry	Condiments and sauces	Semi-solid	Current study	25	4	91 ± 40	22 ± 9	17 ± 7	546	129
Sandwich meat chicken	Meat and meat products	Soft-solid	Current study	30	5	92 ± 25	22 ± 6	18 ± 5	520	124
Coffee cappuccino instant	Non-alcoholic beverages	Liquid	Current study	125	4	92 ± 34	22 ± 8	59 ± 22	156	37
Mandarins	Fruits, nuts and olives	Soft-solid	Current study	50	5	93 ± 44	22 ± 10	48 ± 23	193	45
Pear without skin	Fruits, nuts and olives	Soft-solid	Current study	50	5	94 ± 36	22 ± 9	40 ± 16	232	55
Apple with skin	Fruits, nuts and olives	Hard-solid	Current study	50	174	96 ± 22	23 ± 5	38 ± 9	254	60
Popcorn popped sweet	Sugar and confectionery	Hard-solid	Current study	13	6	99 ± 35	24 ± 8	6 ± 2	1649	389
Bread wholemeal	Cereals and cereal products	Soft-solid	Current study	35	174	99 ± 27	24 ± 6	10 ± 3	990	234
Strawberries	Fruits, nuts and olives	Soft-solid	Current study	50	5	99 ± 55	24 ± 13	81 ± 45	123	29
Cocktail snacks Nibbits	Cereals and cereal products	Hard-solid	Current study	12	5	100 ± 31	24 ± 7	5 ± 2	2021	482
Soup thickened with vegetables	Soups, bouillon	Semi-solid	Previous study	50	4	104 ± 30	25 ± 7	70 ± 21	148	35

Low fat margarine on wholemeal bread	Cereals and cereal products	Soft-solid	Current study	40	5	106 ± 51	25 ± 12	10 ± 5	1047	249
Bread multigrain with seeds	Cereals and cereal products	Soft-solid	Current study	35	5	107 ± 23	25 ± 6	10 ± 2	1099	261
Cabbage red with apple pieces frozen boiled	Vegetables	Soft-solid	Current study	50	5	108 ± 46	26 ± 11	44 ± 19	247	59
Tomato juice	Non-alcoholic beverages	Liquid	Current study	125	5	109 ± 35	26 ± 8	160 ± 51	68	16
Japanese rice cracker mix	Cereals and cereal products	Hard-solid	Current study	25	5	111 ± 20	26 ± 5	7 ± 1	1645	387
Prawn crackers natural	Cereals and cereal products	Hard-solid	Current study	12	5	111 ± 42	26 ± 10	5 ± 2	2158	516
Sauce tomato readymade	Condiments and sauces	Semi-solid	Current study	25	5	111 ± 36	26 ± 9	44 ± 14	253	61
Toast	Cereals and cereal products	Hard-solid	Current study	17	5	112 ± 32	27 ± 8	7 ± 2	1522	359
Peas fresh boiled	Vegetables	Soft-solid	Previous study	50	4	113 ± 71	27 ± 17	39 ± 24	291	69
Soup clear with meat vegetables and noodles	Soups, bouillon	Semi-solid	Current study	75	5	114 ± 55	27 ± 13	66 ± 32	173	41
Pork fillet	Meat and meat products	Soft-solid	Current study	50	5	117 ± 54	28 ± 13	18 ± 8	661	157
Ice cream dairy cream based	Sugar and confectionery	Semi-solid	Previous study	50	4	117 ± 58	28 ± 14	14 ± 7	856	205
Carrot boiled	Vegetables	Soft-solid	Previous study	50	3	119 ± 86	28 ± 21	89 ± 65	133	32
Orange	Fruits, nuts and olives	Soft-solid	Current study	50	5	120 ± 50	29 ± 12	56 ± 23	215	51
Olives ripe in brine	Fruits, nuts and olives	Hard-solid	Current study	25	5	121 ± 36	29 ± 8	18 ± 5	663	162
Cod prepared in microwave oven	Fish and shellfish	Soft-solid	Current study	50	4	123 ± 34	30 ± 8	30 ± 8	414	98
Beans brown tinned	Legumes	Soft-solid	Current study	50	5	129 ± 29	31 ± 7	28 ± 6	460	109
Soup vegetable readymade	Soups, bouillon	Semi-solid	Previous study	50	4	132 ± 57	32 ± 14	67 ± 29	196	47
Bread white water based	Cereals and cereal products	Soft-solid	Current study	35	5	133 ± 25	32 ± 6	13 ± 2	1046	247
Ham shoulder medium fat boiled	Meat and meat products	Soft-solid	Current study	40	5	139 ± 34	33 ± 8	25 ± 6	556	133
Ham shoulder medium fat boiled on wholemeal bread	Cereals and cereal products	Soft-solid	Current study	60	5	140 ± 46	33 ± 11	16 ± 5	861	205
Biscuit salted average	Cereals and cereal products	Hard-solid	Current study	25	5	140 ± 9	33 ± 2	7 ± 0	1975	472
Winegum/fruitgum	Sugar and confectionery	Soft-solid	Current study	20	5	140 ± 33	33 ± 8	10 ± 2	1358	320

Rice brown boiled	Cereals and cereal products	Soft-solid	Previous study	50	3	142 ± 75	34 ± 18	26 ± 14	555	131
Yoghurt low fat	Dairy products	Semi-solid	Current study	75	5	146 ± 25	35 ± 6	94 ± 16	156	37
Spinach creamed frozen boiled	Vegetables	Semi-solid	Current study	50	5	153 ± 34	37 ± 8	51 ± 11	303	73
Roll white hard	Cereals and cereal products	Hard-solid	Current study	35	4	153 ± 42	37 ± 10	13 ± 4	1173	277
Pineapple in syrup	Fruits, nuts and olives	Hard-solid	Current study	40	5	155 ± 36	37 ± 9	53 ± 12	291	69
Roll brown hard	Cereals and cereal products	Hard-solid	Current study	35	5	155 ± 16	37 ± 4	13 ± 1	1177	278
Ice cream stracciatella	Sugar and confectionery	Semi-solid	Current study	50	5	156 ± 34	37 ± 8	16 ± 3	991	237
Grapes with skin	Fruits, nuts and olives	Hard-solid	Current study	50	5	156 ± 42	37 ± 10	47 ± 13	331	78
Muesli with fruit	Cereals and cereal products	Hard-solid	Current study	25	5	156 ± 44	37 ± 10	10 ± 3	1505	357
Crisps potato light unflavored	Cereals and cereal products	Hard-solid	Current study	25	5	156 ± 54	37 ± 13	8 ± 3	2051	490
Fromage frais low fat	Dairy products	Semi-solid	Current study	75	4	157 ± 87	38 ± 21	63 ± 35	248	58
Cheese Mozzarella on wholemeal bread	Cereals and cereal products	Soft-solid	Current study	55	5	158 ± 70	38 ± 17	15 ± 7	1026	244
Peaches in syrup	Fruits, nuts and olives	Soft-solid	Current study	50	5	158 ± 100	38 ± 24	60 ± 38	263	62
Toffees	Sugar and confectionery	Soft-solid	Previous study	50	3	158 ± 58	38 ± 14	9 ± 3	1796	428
Roll white soft	Cereals and cereal products	Soft-solid	Current study	25	5	163 ± 59	39 ± 14	15 ± 5	1108	262
Liquorice Dutch type salted	Sugar and confectionery	Soft-solid	Current study	20	5	163 ± 51	39 ± 12	11 ± 4	1418	334
Raisins dried	Fruits, nuts and olives	Soft-solid	Current study	30	6	163 ± 38	39 ± 9	12 ± 3	1382	326
Biscuit sweet	Cakes	Hard-solid	Current study	13	5	166 ± 51	40 ± 12	9 ± 3	1841	437
Sandwich meat chicken on wholemeal bread	Cereals and cereal products	Soft-solid	Current study	60	5	168 ± 22	40 ± 5	20 ± 3	849	202
Bread pita white	Cereals and cereal products	Hard-solid	Current study	25	5	169 ± 34	40 ± 8	16 ± 3	1040	245
Banana	Fruits, nuts and olives	Soft-solid	Previous study	50	25	171 ± 55	41 ± 13	43 ± 14	401	95
Margarine 80% fat on wholemeal bread	Cereals and cereal products	Soft-solid	Current study	40	5	171 ± 55	41 ± 13	14 ± 4	1237	295
Eggs chicken boiled	Eggs and egg products	Soft-solid	Previous study	50	24	173 ± 72	41 ± 17	32 ± 13	535	128
Roll brown soft	Cereals and cereal products	Soft-solid	Current study	30	5	175 ± 35	42 ± 8	16 ± 3	1087	258
Crackers cream	Cereals and cereal	Hard-solid	Previous study	50	3	175 ± 19	42 ± 5	9 ± 1	1973	469

	products									
Pineapple	Fruits, nuts and olives	Hard-solid	Previous study	50	2	176 ± 112	42 ± 27	73 ± 46	242	57
Wrap/Tortilla	Cereals and cereal products	Soft-solid	Current study	40	5	176 ± 61	42 ± 15	13 ± 5	1349	320
Beans baked in tomato sauce tinned	Legumes	Soft-solid	Current study	50	4	176 ± 75	42 ± 18	45 ± 19	393	93
Herring salted	Fish and shellfish	Soft-solid	Current study	60	5	176 ± 80	42 ± 19	24 ± 11	720	172
Biscuit sponge fingers	Cakes	Hard-solid	Current study	12	5	177 ± 34	42 ± 8	10 ± 2	1702	402
Crisps tortilla unflavored	Cereals and cereal products	Hard-solid	Current study	25	5	178 ± 34	43 ± 8	9 ± 2	2039	487
Biscuit fruit	Cakes	Hard-solid	Current study	15	5	179 ± 18	43 ± 4	11 ± 1	1662	393
Cheese Gouda 48+	Dairy products	Soft-solid	Previous study	50	24	179 ± 67	43 ± 16	12 ± 4	1529	369
Potatoes mashed	Potatoes and other tubers	Soft-solid	Previous study	50	3	183 ± 152	44 ± 36	52 ± 44	349	83
Muesli crunchy	Cereals and cereal products	Hard-solid	Current study	25	5	185 ± 32	44 ± 8	10 ± 2	1841	438
Chocolate plain	Sugar and confectionery	Hard-solid	Current study	35	5	185 ± 78	44 ± 19	8 ± 4	2209	531
Cake sponge Dutch Eierkoek	Cakes	Soft-solid	Current study	15	5	185 ± 36	44 ± 9	14 ± 3	1314	310
Bread ciabatta no filling	Cereals and cereal products	Hard-solid	Current study	50	5	188 ± 19	45 ± 5	17 ± 2	1079	255
Biscuit brown/wholemeal	Cakes	Hard-solid	Current study	17	5	190 ± 25	45 ± 6	10 ± 1	1928	459
Potato crisps oven baked	Cereals and cereal products	Hard-solid	Current study	25	5	191 ± 96	46 ± 23	11 ± 6	1731	411
Beer pilsner	Alcoholic beverages	Liquid	Previous study	125	24	198 ± 128	47 ± 31	106 ± 69	187	45
Soup legume based readymade	Soups, bouillon	Semi-solid	Current study	75	5	199 ± 47	47 ± 11	59 ± 14	337	80
Hot chocolate	Dairy products	Liquid	Current study	125	5	200 ± 139	48 ± 33	71 ± 50	280	66
Pork schnitzel breaded	Meat and meat products	Hard-solid	Current study	50	4	204 ± 49	49 ± 12	26 ± 6	788	188
Beef steak tartare	Meat and meat products	Soft-solid	Current study	40	5	204 ± 52	49 ± 12	26 ± 7	782	186
Spring roll fried	Miscellaneous	Hard-solid	Current study	25	5	204 ± 52	49 ± 12	27 ± 7	757	181
Cashew nuts unsalted	Fruits, nuts and olives	Hard-solid	Current study	50	5	206 ± 81	49 ± 19	8 ± 3	2552	615
Sandwich spread original on wholemeal bread	Condiments and sauces	Soft-solid	Current study	55	5	207 ± 48	49 ± 12	21 ± 5	975	235
Sauce for chips 25% oil	Condiments and sauces	Semi-solid	Current study	25	5	213 ± 243	51 ± 58	17 ± 20	1226	296
Yoghurt half fat	Dairy products	Semi-solid	Current study	75	174	215 ± 55	51 ± 13	102 ± 26	212	50
Tortellini boiled	Cereals and cereal products	Soft-solid	Current study	50	5	216 ± 80	52 ± 19	33 ± 12	656	155
Cheese 30+ on wholemeal	Cereals and cereal	Soft-solid	Current study	60	5	217 ± 47	52 ± 11	20 ± 4	1094	261

bread	products									
White fish fillet in batter deep-fried	Fish and shellfish	Hard-solid	Current study	50	5	221 ± 88	53 ± 21	25 ± 10	883	211
Potato slices fried	Potatoes and other tubers	Soft-solid	Current study	50	5	223 ± 51	53 ± 12	28 ± 7	789	189
Cultured confetti fruit-flavored on wholemeal bread	Cereals and cereal products	Hard-solid	Current study	55	4	224 ± 110	54 ± 26	19 ± 9	1192	282
Biscuit fortified with currants (LigaEvergreen)	Cakes	Hard-solid	Current study	20	4	229 ± 93	55 ± 22	14 ± 6	1643	390
Yoghurt vanilla half fat	Dairy products	Semi-solid	Current study	75	5	231 ± 100	55 ± 24	70 ± 30	330	78
Bun wholemeal with muesli	Cereals and cereal products	Hard-solid	Current study	35	5	231 ± 32	55 ± 8	19 ± 3	1221	290
Cheese spread 48+ on wholemeal bread	Cereals and cereal products	Soft-solid	Current study	55	5	234 ± 57	56 ± 14	23 ± 6	1032	246
Chicken fillet	Meat and meat products	Soft-solid	Previous study	50	4	234 ± 134	56 ± 32	35 ± 20	667	158
Biscuit spiced Speculaas	Cakes	Hard-solid	Previous study	50	25	235 ± 71	56 ± 17	12 ± 4	1993	475
Chocolate flakes plain on wholemeal bread	Cereals and cereal products	Hard-solid	Current study	55	4	236 ± 80	56 ± 19	19 ± 6	1245	296
Beef steak tartare spiced (filet americain) on wholemeal bread	Cereals and cereal products	Soft-solid	Current study	55	5	238 ± 58	57 ± 14	24 ± 6	1009	240
Biscuit fortified (Liga Milkbreak)	Cakes	Hard-solid	Current study	20	5	238 ± 38	57 ± 9	13 ± 2	1863	444
Cake Dutch spiced (Ontbijtkoek)	Cakes	Soft-solid	Previous study	50	25	239 ± 77	57 ± 18	18 ± 6	1305	308
Candy bar Snickers	Sugar and confectionery	Hard-solid	Current study	37	5	239 ± 79	57 ± 19	12 ± 4	2029	484
Cheese 30+	Dairy products	Soft-solid	Current study	50	5	241 ± 213	58 ± 51	20 ± 18	1203	289
Salami sausage saveloy on wholemeal bread	Cereals and cereal products	Soft-solid	Current study	60	5	245 ± 138	59 ± 33	21 ± 12	1189	284
Marsh mallows	Sugar and confectionery	Soft-solid	Current study	25	5	249 ± 109	60 ± 26	18 ± 8	1402	330
Peanuts coated	Fruits, nuts and olives	Hard-solid	Current study	25	5	251 ± 109	60 ± 26	11 ± 5	2251	540
Cheese Brie 60+ on wholemeal bread	Cereals and cereal products	Soft-solid	Current study	60	5	252 ± 27	60 ± 7	21 ± 2	1185	283
Crisps potato	Cereals and cereal products	Hard-solid	Current study	25	4	252 ± 72	60 ± 17	11 ± 3	2261	542
Salmon prepared in microwave oven	Fish and shellfish	Soft-solid	Current study	40	5	254 ± 75	61 ± 18	28 ± 8	918	220
Minced beef/pork shallow	Meat and meat products	Soft-solid	Current study	50	5	254 ± 165	61 ± 39	19 ± 12	1322	317

fried										
Cake Dutch spiced (Ontbijtkoek) wholemeal	Cakes	Soft-solid	Current study	30	5	256 ± 77	61 ± 18	20 ± 6	1303	308
Tuna in oil tinned	Fish and shellfish	Soft-solid	Current study	50	5	256 ± 58	61 ± 14	30 ± 7	862	206
Cheese cream soft (Boursin) on wholemeal bread	Cereals and cereal products	Soft-solid	Current study	50	5	257 ± 53	62 ± 13	21 ± 4	1202	287
Crisps potato light flavored	Cereals and cereal products	Hard-solid	Previous study	50	5	258 ± 138	62 ± 33	13 ± 7	2015	481
Biscuit Dutch (Krakeling)	Cakes	Hard-solid	Current study	17	5	263 ± 53	63 ± 13	13 ± 3	2075	496
Cheese Mozzarella	Dairy products	Soft-solid	Current study	35	5	264 ± 117	63 ± 28	24 ± 11	1089	262
Liquorice Dutch sweet	Sugar and confectionery	Soft-solid	Previous study	50	3	273 ± 92	65 ± 22	19 ± 6	1437	338
Chips oven frozen prepared	Potatoes and other tubers	Hard-solid	Previous study	50	3	276 ± 205	66 ± 49	22 ± 17	1231	293
Syrup apple on wholemeal bread	Cereals and cereal products	Soft-solid	Current study	55	4	276 ± 92	66 ± 22	26 ± 9	1061	251
Hamburger prepared	Meat and meat products	Soft-solid	Current study	40	5	277 ± 75	66 ± 18	26 ± 7	1062	255
Liquorice allsorts	Sugar and confectionery	Soft-solid	Current study	20	5	278 ± 57	66 ± 14	17 ± 3	1665	394
Kiwi fruit green	Fruits, nuts and olives	Soft-solid	Current study	50	5	278 ± 80	67 ± 19	97 ± 28	286	68
Cheese Brie 60+	Dairy products	Soft-solid	Current study	50	6	279 ± 121	67 ± 29	18 ± 8	1529	369
Pasta wholemeal boiled	Cereals and cereal products	Soft-solid	Previous study	50	2	286 ± 26	68 ± 6	52 ± 5	555	131
Chocolate confetti plain on wholemeal bread	Cereals and cereal products	Hard-solid	Current study	55	6	287 ± 99	69 ± 24	23 ± 8	1251	297
Bun currant/raisin	Cereals and cereal products	Soft-solid	Current study	35	6	293 ± 65	70 ± 16	26 ± 6	1134	268
Sausage luncheon meat on wholemeal bread	Cereals and cereal products	Soft-solid	Current study	60	5	294 ± 75	70 ± 18	27 ± 7	1102	263
Croissant	Cakes	Hard-solid	Current study	27	5	299 ± 89	71 ± 21	18 ± 5	1684	403
Jam on wholemeal bread	Cereals and cereal products	Soft-solid	Current study	55	5	301 ± 58	72 ± 14	29 ± 6	1026	243
Peanut butter on wholemeal bread	Cereals and cereal products	Soft-solid	Current study	55	5	303 ± 105	72 ± 25	20 ± 7	1481	355
Rice white boiled	Cereals and cereal products	Soft-solid	Previous study	50	4	304 ± 232	73 ± 55	49 ± 37	621	146
Mayonnaise	Condiments and sauces	Semi-solid	Current study	25	3	307 ± 171	73 ± 41	11 ± 6	2733	664
Spread chocolate hazelnut on wholemeal bread	Cereals and cereal products	Soft-solid	Current study	55	5	307 ± 146	73 ± 35	22 ± 11	1376	328

Chicken nuggets prepared in oven	Meat and meat products	Hard-solid	Current study	40	5	307 ± 115	73 ± 27	28 ± 11	1083	259
Yoghurt low fat with fruit	Dairy products	Semi-solid	Current study	75	5	308 ± 73	74 ± 18	103 ± 24	300	71
Chips fried in liquid frying fat	Potatoes and other tubers	Hard-solid	Current study	50	5	308 ± 41	74 ± 10	24 ± 3	1300	311
Sausage luncheon meat	Meat and meat products	Soft-solid	Current study	40	5	309 ± 172	74 ± 41	24 ± 13	1280	309
Yoghurt full fat	Dairy products	Semi-solid	Current study	75	6	319 ± 112	76 ± 27	132 ± 46	242	58
Peanut sauce ready to eat	Condiments and sauces	Semi-solid	Current study	25	6	321 ± 95	77 ± 23	31 ± 9	1039	249
Liver pate sausage on wholemeal bread	Cereals and cereal products	Soft-solid	Current study	55	5	322 ± 117	77 ± 28	29 ± 11	1097	262
Sausage pork (Braadworst)	Meat and meat products	Soft-solid	Current study	50	5	333 ± 151	80 ± 36	33 ± 15	1025	246
Mousse chocolate	Dairy products	Semi-solid	Current study	75	4	334 ± 130	80 ± 31	44 ± 17	763	182
Salami sausage saveloy	Meat and meat products	Soft-solid	Current study	50	6	334 ± 204	80 ± 49	22 ± 13	1541	372
Bread white with sugar (Suikerbrood)	Cereals and cereal products	Soft-solid	Current study	35	5	336 ± 66	80 ± 16	26 ± 5	1291	305
Buttermilk	Dairy products	Liquid	Previous study	125	25	336 ± 308	80 ± 74	262 ± 241	128	30
Energy drink (Red Bull)	Non-alcoholic beverages	Liquid	Current study	125	5	338 ± 195	81 ± 47	181 ± 104	187	44
Chocolate chip cookie	Cakes	Hard-solid	Current study	38	5	341 ± 39	81 ± 9	16 ± 2	2120	506
Meringue cake (Bokkenpootje)	Cakes	Hard-solid	Current study	25	5	342 ± 50	82 ± 12	18 ± 3	1903	454
Chocolates filled/Belgium chocolate	Sugar and confectionery	Soft-solid	Current study	50	5	348 ± 93	83 ± 22	16 ± 4	2136	512
Candy bar Twix	Sugar and confectionery	Hard-solid	Current study	20	5	354 ± 108	85 ± 26	17 ± 5	2071	495
Biscuit Dutch shortbread sprits	Cakes	Hard-solid	Current study	25	5	355 ± 53	85 ± 13	16 ± 2	2205	527
Sausage (Frikandel) deep-fried	Meat and meat products	Soft-solid	Current study	37	5	357 ± 166	85 ± 40	34 ± 16	1049	252
Waffle Luikse	Cakes	Soft-solid	Current study	30	5	358 ± 228	85 ± 54	19 ± 12	1868	446
Candy bar (Milky Way)	Sugar and confectionery	Hard-solid	Current study	30	5	368 ± 147	88 ± 35	20 ± 8	1883	448
Peanuts salted	Fruits, nuts and olives	Hard-solid	Previous study	50	4	370 ± 173	88 ± 41	14 ± 7	2586	624
Salmon smoked	Fish and shellfish	Soft-solid	Previous study	50	3	372 ± 249	89 ± 59	48 ± 32	771	185
Yoghurt drink with sweetener	Dairy products	Liquid	Previous study	50	4	375 ± 312	90 ± 75	307 ± 256	122	29
Eclair with whipped cream filling	Cakes	Soft-solid	Current study	40	5	378 ± 157	90 ± 38	32 ± 13	1186	286
Bacon rasher	Meat and meat products	Soft-solid	Current study	40	5	381 ± 102	91 ± 24	21 ± 6	1804	435
Chocolate bar milk with nuts	Sugar and confectionery	Hard-solid	Current study	25	5	386 ± 168	92 ± 40	16 ± 7	2342	562

Croissant with ham and cheese	Miscellaneous	Hard-solid	Current study	45	5	401 ± 125	96 ± 30	27 ± 9	1460	350
Croquette meat deep-fried in liquid fat	Miscellaneous	Hard-solid	Current study	40	5	410 ± 84	98 ± 20	36 ± 7	1139	273
Pudding airy	Dairy products	Semi-solid	Current study	75	6	413 ± 147	99 ± 35	43 ± 15	967	231
Sausage frankfurter tinned	Meat and meat products	Soft-solid	Current study	40	4	415 ± 112	99 ± 27	50 ± 13	837	201
Cashew nuts salted	Fruits, nuts and olives	Hard-solid	Previous study	50	2	438 ± 373	105 ± 89	17 ± 15	2552	615
Roll bapao	Miscellaneous	Soft-solid	Current study	60	5	440 ± 128	105 ± 30	38 ± 11	1152	273
Ice tea (non-sparkling)	Non-alcoholic beverages	Liquid	Current study	125	5	454 ± 105	109 ± 25	344 ± 79	132	31
Snack sausage roll with bread dough pastry	Miscellaneous	Hard-solid	Current study	40	5	459 ± 55	110 ± 13	29 ± 3	1596	382
Sausage smoked traditional cooked	Meat and meat products	Soft-solid	Current study	50	5	463 ± 135	111 ± 32	36 ± 10	1287	311
Chocolate milk	Sugar and confectionery	Hard-solid	Previous study	50	4	464 ± 184	111 ± 44	20 ± 8	2286	548
Custard vanilla full fat	Dairy products	Semi-solid	Previous study	50	4	472 ± 213	113 ± 51	121 ± 55	390	93
Cream slice Dutch (Tompouce)	Cakes	Hard-solid	Current study	45	5	472 ± 138	113 ± 33	39 ± 11	1208	288
Pancake prepared with margarine	Cakes	Soft-solid	Current study	65	5	478 ± 211	114 ± 50	58 ± 26	826	196
Apple sauce	Fruits, nuts and olives	Semi-solid	Previous study	50	3	479 ± 307	115 ± 73	147 ± 95	325	77
Cream whipped with added sugar	Dairy products	Semi-solid	Current study	25	5	479 ± 212	115 ± 51	33 ± 15	1453	351
Custard chocolate full fat	Dairy products	Semi-solid	Current study	75	4	512 ± 159	122 ± 38	122 ± 38	419	100
Apple pie Dutch with shortbread	Cakes	Soft-solid	Current study	50	5	531 ± 104	127 ± 25	50 ± 10	1064	253
Ice tea (sparkling)	Non-alcoholic beverages	Liquid	Current study	125	4	546 ± 295	130 ± 70	414 ± 223	132	31
Fromage frais half fat with fruit	Dairy products	Semi-solid	Current study	50	5	546 ± 185	131 ± 44	104 ± 35	523	124
Dairy spread plain/herbs on wholemeal bread	Cereals and cereal products	Soft-solid	Current study	55	5	549 ± 802	131 ± 192	54 ± 78	1022	244
Apple turnover with puff pastry	Cakes	Hard-solid	Current study	55	5	552 ± 146	132 ± 35	37 ± 10	1506	361
Milk semi-skimmed	Dairy products	Liquid	Previous study	125	25	555 ± 383	133 ± 92	289 ± 200	192	46
Cupcake iced	Cakes	Soft-solid	Current study	38	5	568 ± 93	136 ± 22	31 ± 5	1833	437
Almond paste filled tarts	Cakes	Soft-solid	Current study	20	5	593 ± 234	142 ± 56	35 ± 14	1697	404
Smoothie fruit	Non-alcoholic beverages	Liquid	Current study	125	5	594 ± 26	142 ± 6	257 ± 11	231	54
Cake wrapped in marzipan and chocolate	Cakes	Soft-solid	Current study	30	4	608 ± 239	145 ± 57	32 ± 13	1900	454

Fruit drink concentrate diluted	Sugar and confectionery	Liquid	Current study	125	5	610 ± 278	146 ± 66	268 ± 122	227	54
Cake without butter	Cakes	Soft-solid	Previous study	50	4	636 ± 303	152 ± 72	34 ± 16	1856	444
Minced meat ball with egg/crumbs	Meat and meat products	Soft-solid	Previous study	50	3	654 ± 109	156 ± 26	58 ± 10	1127	270
Breakfast drink (Goede Morgen original)	Dairy products	Liquid	Current study	125	5	673 ± 135	161 ± 32	271 ± 55	248	59
Waffle syrup	Cakes	Soft-solid	Previous study	50	3	685 ± 387	164 ± 93	35 ± 20	1936	461
Snack sausage roll puff pastry	Miscellaneous	Hard-solid	Current study	37	5	761 ± 275	182 ± 66	51 ± 19	1484	356
Milk skimmed	Dairy products	Liquid	Current study	125	5	786 ± 97	188 ± 23	527 ± 65	149	35
Yoghurt drink	Dairy products	Liquid	Current study	125	5	793 ± 296	190 ± 71	330 ± 123	240	57
Milk whole	Dairy products	Liquid	Current study	125	5	961 ± 567	230 ± 136	373 ± 220	258	62
Juice orange pasteurized	Non-alcoholic beverages	Liquid	Current study	125	5	971 ± 598	232 ± 143	511 ± 315	190	45
Juice orange freshly squeezed	Non-alcoholic beverages	Liquid	Current study	125	5	1004 ± 468	240 ± 112	483 ± 225	208	49
Milk chocolate-flavored semi-skimmed	Dairy products	Liquid	Current study	125	5	1044 ± 307	249 ± 73	319 ± 94	327	77
Juice apple	Non-alcoholic beverages	Liquid	Previous study	50	2	1243 ± 175	297 ± 42	641 ± 90	194	46
Breakfast drink (HeroFruitontbijt)	Non-alcoholic beverages	Liquid	Previous study	50	4	1379 ± 1095	330 ± 262	595 ± 472	232	55
Milk chocolate-flavored full fat	Dairy products	Liquid	Previous study	50	4	1766 ± 1388	422 ± 332	471 ± 370	375	89

Table S2. Frequency (*n* (%)) of food groups in the eating rate and energy intake rate quartiles (*n* = 240).

	**Eating rate (g/min)**					**Energy intake rate (kJ/min)[1]**				
	**Quartile 1 2–16 g/min (*n* = 60)**	**Quartile 2 16–26 g/min (*n* = 60)**	**Quartile 3 26–50 g/min (*n* = 60)**	**Quartile 4 50–641 g/min (*n* = 60)**	p^2	**Quartile 1 0–112 kJ/min (*n* = 60)**	**Quartile 2 113–204 kJ/min (*n* = 60)**	**Quartile 3 204–333 kJ/min (*n* = 60)**	**Quartile 4 334–1766 kJ/min (*n* = 60)**	p^2
**Food groups**					<0.0001					<0.0001
Potatoes	0 (0.0)	3 (5.0)	2 (3.3)	1 (1.7)		2 (3.3)	1 (1.7)	3 (5.0)	0 (0.0)	
Vegetables	2 (3.3)	5 (8.3)	13 (21.7)	4 (6.7)		21 (35.0)	3 (5.0)	0 (0.0)	0 (0.0)	
Legumes	0 (0.0)	0 (0.0)	2 (3.3)	0 (0)		0 (0)	2 (3.3)	0 (0)	0 (0)	
Fruits, nuts and olives	4 (6.7)	3 (5.0)	6 (10.0)	7 (11.7)		6 (10.0)	8 (13.3)	3 (5.0)	3 (5.0)	
Dairy products	1 (1.7)	3 (5.0)	3 (5.0)	19 (31.7)		0 (0.0)	4 (6.7)	7 (11.7)	15 (25.0)	
Cereals and cereal products	30 (50.0)	19 (31.7)	5 (8.3)	2 (3.3)		12 (20.0)	20 (33.3)	22 (36.7)	2 (3.3)	
Meat and meat products	1 (1.7)	8 (13.3)	8 (13.3)	1 (1.7)		2 (3.3)	4 (6.7)	6 (10.0)	6 (10.0)	
Fish and shellfish	0 (0.0)	2 (3.3)	4 (6.7)	0 (0.0)		0 (0.0)	2 (3.3)	3 (5.0)	1 (1.7)	
Eggs and egg products	0 (0.0)	0 (0.0)	1 (1.7)	0 (0.0)		0 (0.0)	1 (1.7)	0 (0.0)	0 (0.0)	
Sugar and confectionery	10 (16.7)	8 (13.3)	0 (0.0)	1 (1.7)		3 (5.0)	6 (10.0)	4 (6.7)	6 (10.0)	
Cakes	11 (18.3)	5 (8.3)	8 (13.3)	2 (3.3)		0 (0.0)	5 (8.3)	7 (11.7)	14 (23.3)	
Non-alcoholic beverages	0 (0.0)	0 (0.0)	0 (0.0)	15 (25.0)		7 (11.7)	0 (0.0)	0 (0.0)	8 (13.3)	
Alcoholic beverages	0 (0.0)	0 (0.0)	0 (0.0)	1 (1.7)		0 (0.0)	1 (1.7)	0 (0.0)	0 (0.0)	
Condiments and sauces	1 (1.7)	4 (6.7)	2 (3.3)	0 (0.0)		3 (5.0)	0 (0.0)	4 (6.7)	0 (0.0)	
Soups, bouillon	0 (0.0)	0 (0.0)	1 (1.7)	6 (10.0)		4 (6.7)	3 (5.0)	0 (0.0)	0 (0.0)	
Snacks	0 (0.0)	0 (0.0)	5 (8.3)	1 (1.7)		0 (0)	0 (0)	1 (1.67	5 (8.3)	

Table S3 Characteristics (mean ± SD) of the foods in the eating rate and energy intake rate quartiles (*n* = 240): eating rate, energy intake rate, food composition and taste.

	Eating rate (g/min)					Energy intake rate (kJ/min) [1]				
	**Quartile 1** 2–16 g/min (*n* = 60)	**Quartile 2** 16–26 g/min (*n* = 60)	**Quartile 3** 26–50 g/min (*n* = 60)	**Quartile 4** 50–641 g/min (*n* = 60)	*p* [2]	**Quartile 1** 0–112 kJ/min (*n* = 60)	**Quartile 2** 113–204 kJ/min (*n* = 60)	**Quartile 3** 204–333 kJ/min (*n* = 60)	**Quartile 4** 334–1766 kJ/min (*n* = 60)	*p* [2]
**Eating rate (g/min)**	11 ± 3	21 ± 3	36 ± 7	192 ± 170	<0.0001	58 ± 106	30 ± 25	29 ± 24	143 ± 173	<0.0001
**Energy intake rate**					<0.0001					<0.0001
kJ/min	162 ± 76	230 ± 108	254 ± 183	398 ± 375		62 ± 36	161 ± 25	262 ± 35	560 ± 278	
kcal/min	39 ± 18	55 ± 26	61 ± 44	95 ± 90		15 ± 9	38 ± 6	63 ± 8	134 ± 66	
**Food composition**										
Energy (kJ/100 g)	1586 ± 545	1144 ± 582	747 ± 5552	285 ± 282	<0.0001	500 ± 633	951 ± 619	1226 ± 539	1086 ± 764	<0.0001
Protein (g/100 g)	9 ± 5	10 ± 8	8 ± 8	3 ± 3	<0.0001	3 ± 5	8 ± 7	10 ± 8	7 ± 7	<0.01
Fat (g/100 g)	14 ± 16	12 ± 12	9 ± 9	2 ± 4	<0.0001	2 ± 5	7 ± 11	14 ± 13	14 ± 13	<0.0001
Carbohydrate (g/100 g)	54 ± 24	30 ± 25	17 ± 17	9 ± 8	<0.0001	21 ± 29	32 ± 27	30 ± 24	26 ± 22	0.34
Mono- and disaccharides (g/100 g)	17 ± 21	14 ± 19	8 ± 11	18 ± 94	0.93	7 ± 16	23 ± 95	12 ± 16	16 ± 16	0.54
Polysaccharides (g/100 g)	37 ± 22	15 ± 14	9 ± 11	3 ± 6	<0.0001	14 ± 24	21 ± 22	19 ± 15	10 ± 12	0.15
Fiber (g/100 g)	4 ± 3	3 ± 2	2 ± 2	1 ± 1	<0.0001	2 ± 2	3 ± 3	3 ± 2	1 ± 1	0.02
Water (g/100 g)	19 ± 23	44 ± 26	64 ± 24	85 ± 14	<0.0001	71 ± 35	49 ± 32	41 ± 24	51 ± 32	<0.001
Sodium (mg/100 g)	361 ± 275	356 ± 300	258 ± 301	87 ± 142	<0.0001	217 ± 302	278 ± 277	333 ± 234	234 ± 309	0.53
**Taste intensity** [3]										
Sweet	22 ± 19	24 ± 23	20 ± 20	23 ± 17	0.87	12 ± 12	18 ± 17	22 ± 20	36 ± 21	<0.0001
Sour	4 ± 6	8 ± 12	9 ± 12	20 ± 18	<0.0001	10 ± 14	9 ± 13	11 ± 16	11 ± 14	0.43
Bitter	2 ± 5	2 ± 3	2 ± 3	6 ± 13	0.01	5 ± 10	3 ± 10	2 ± 2	3 ± 3	0.09
Umami	5 ± 8	8 ± 9	11 ± 11	5 ± 9	0.37	8 ± 11	6 ± 9	9 ± 10	5 ± 9	0.41
Salt	18 ± 15	18 ± 13	18 ± 18	10 ± 15	<0.01	12 ± 15	17 ± 16	21 ± 14	14 ± 16	0.30
Fat	22 ± 18	34 ± 19	29 ± 20	20 ± 15	0.28	12 ± 10	21 ± 15	36 ± 17	37 ± 19	<0.0001

[1] Energy intake rate-quartiles (kcal/min); Quartile 1 = 0–27 kcal/min, Quartile 2 = 27–49 kcal/min, Quartile 3 = 49–80 kcal/min, Quartile 4 = 80–422 kcal/min; [2] *p*-value linear trend; [3] *n* = 225.

Table S4 Energy intake rate (kcal/min) of foods relative to the other foods within the food group.

**Food group**	**Energy intake rate (kcal/min) relative to food group**		
	**Low**	**Medium**	**High**
**Potatoes (*n* = 6)**			
Description	**Boiled potatoes [1] (*n* = 2)**		**Mashed and (deep-)fried potatoes (*n* = 4)**
Energy intake rate [1]	18 (15–21) kcal/min		59 (44–74) kcal/min
Eating rate [1]	23 (18–28) g/min		32 (22–52) g/min
Energy density [1]	79 (74–83) kcal/100 g		219 (83–311) kcal/100 g
**Vegetables (*n* = 24)**			
Description	**Raw vegetables [1] (*n* = 5)**	**Boiled [1] and pickled vegetables (*n* = 17)**	**Vegetables with added energy (*n* = 2)**
Energy intake rate [1]	7 (2–17) kcal/min	11 (2–28) kcal/min	31 (26–37) kcal/min
Eating rate [1]	36 (12–76) g/min	37 (13–89) g/min	48 (44–51) g/min
Energy density [1]	19 (12–33) kcal/100 g	29 (17–69) kcal/100 g	66 (59–73) kcal/100 g
**Legumes (*n* = 2)**			
Description	**Tinned brown beans [1] (*n* = 1)**		**Tinned beans in tomato sauce (*n* = 1)**
Energy intake rate [1]	31 kcal/min		42 kcal/min
Eating rate [1]	28 g/min		45 g/min
Energy density [1]	109 kcal/100 g		93 kcal/100 g
**Fruits, nuts and olives (*n* = 20)**			
Description	**Fruit (excluding soft fruit) [1] (*n* = 8)**	**Olives, conserved fruit and soft fruit [1] (*n* = 7)**	**Nuts [2], apple sauce (*n* = 5)**
Energy intake rate [1]	26 (14–42) kcal/min	39 (24–66) kcal/min	83 (49–114) kcal/min
Eating rate [1]	46 (26–73) g/min	52 (12–97) g/min	39 (8–147) g/min
Energy density [1]	57 (45–78) kcal/100 g	116 (29–326) kcal/100 g	494 (77–624) kcal/100 g
**Dairy products (*n* = 26)**			
Description	**Plain yoghurt and fromage frais [2], cheese [2] (*n* = 8)**	**Deserts other than plain yoghurt or fromage frais (*n* = 8)**	**Dairy drinks [2] (*n* = 10)**
Energy intake rate [1]	54 (35–76) kcal/min	98 (55–130) kcal/min	179 (48–422) kcal/min
Eating rate [1]	58 (12–132) g/min	80 (33–122) g/min	322 (71–527) g/min

Energy density [1]	187 (37–369) kcal/100 g	154 (71–351) kcal/100 g	55 (29–89) kcal/100 g
**Cereals and cereal products (*n* = 56)**			
Description	**Hard and dry products, plain bread slices [2] (*n* = 23)**		**Other (e.g. bread with topping, buns, pasta, rice) [2] (*n* = 33)**
Energy intake rate [1]	33 (9–62) kcal/min		58 (25–131) kcal/min
Eating rate [1]	9 (2–13) g/min		24 (10–54) g/min
Energy density [1]	389 (234–542) kcal/100 g		254 (131–355) kcal/100 g
**Meat and meat products (*n* = 18)**			
Description	**Fresh meat (excluding minced meat) [2] (*n* = 2)**		**Minced meat [2] and processed meat (*n* = 16)**
Energy intake rate [1]	42 (28–56) kcal/min		72 (17–156) kcal/min
Eating rate [1]	27 (18–35) g/min		29 (13–58) g/min
Energy density [1]	158 (157–158) kcal/100 g		250 (124–435) kcal/100 g
**Fish and shellfish (*n* = 6)**			
Description		**Fish and fish products [1] (*n* = 6)**	
Energy intake rate [1]		56 (29–89) kcal/min	
Eating rate [1]		31 (24–48) g/min	
Energy density [1]		182 (98–220) kcal/100 g	
**Eggs and egg products (*n* = 1)**			
Description		**Boiled egg [1] (*n* = 1)**	
Energy intake rate [1]		41 kcal/min	
Eating rate [1]		32 g/min	
Energy density [1]		128 kcal/100 g	
**Sugar and confectionery (*n* = 19)**			
Description	**Hard confectionary (non-chocolate), ice cream (*n* = 5)**	**Soft confectionary (non-chocolate) (*n* = 6)**	**Chocolate, candy bars, fruit drink (*n* = 8)**
Energy intake rate [1]	24 (15–37) kcal/min	50 (33–66) kcal/min	88 (44–146) kcal/min
Eating rate [1]	9 (4–16) g/min	14 (9–19) g/min	47 (8–268) g/min
Energy density [1]	321 (205–394) kcal/100 g	357 (320–428) kcal/100 g	454 (54–562) kcal/100 g
**Cakes (*n* = 26)**			
Description	**Dry cakes, biscuits (*n* = 15)**		**Cakes, pies, pastries, puddings (non-milk based) (*n* = 11)**
Energy intake rate [1]	76 (40–164) kcal/min		104 (57–152) kcal/min

Eating rate [1]	17 (9–35) g/min		33 (18–58) g/min
Energy density [1]	443 (310–527) kcal/100 g		336 (196–446) kcal/100 g
**Non-alcoholic beverages (*n* = 15)**			
Description	**Non– and very low caloric beverages [3] (*n* = 5)**		**Caloric beverages (*n* = 10)**
Energy intake rate [1]	0 (0–1) kcal/min		161 (22–330) kcal/min
Eating rate [1]	334 (56–635) g/min		365 (59–641) g/min
Energy density [1]	0 (0–1) kcal/100 g		41 (16–55) kcal/100 g
**Alcoholic beverages (*n* = 1)**			
Description		**Pilsner beer (*n* = 1)**	
Energy intake rate [1]		47 kcal/min	
Eating rate [1]		106 g/min	
Energy density [1]		45 kcal/100 g	
**Condiments and sauces (*n* = 7)**			
Description	**Tomato sauces (*n* = 3)**		**Mayonnaises and similar (*n* = 4)**
Energy intake rate [1]	22 (19–27) kcal/min		63 (49–77) kcal/min
Eating rate [1]	28 (17–44) g/min		20 (11–31) g/min
Energy density [1]	89 (61–129) kcal/100 g		361 (235–664) kcal/100 g
**Soups, bouillon (*n* = 7)**			
Description	**Soup from cube or package (*n* = 3)**		**Soup with more (semi-) solid components (*n* = 4)**
Energy intake rate [1]	12 (9–18) kcal/min		33 (25–48) kcal/min
Eating rate [1]	89 (41–174) g/min		66 (59–70) g/min
Energy density [1]	20 (5–33) kcal/100 g		51 (35–80) kcal/100 g
**Snacks (*n* = 6)**			
Description	**Spring roll fried (*n* = 1)**		**Other warm savory snacks (*n* = 5)**
Energy intake rate [1]	49 kcal/min		118 (96–182) kcal/min
Eating rate [1]	27 g/min		36 (27–51) g/min
Energy density [1]	181 kcal/100 g		327 (273–382) kcal/100 g

[1] Recommended foods. [2] Both recommended and not recommended foods.
